# Mitochondrial activity disruption and local muscle damage induced in mice by *Scolopendra polymorpha* venom

**DOI:** 10.1590/1678-9199-JVATITD-2019-0079

**Published:** 2020-05-29

**Authors:** Judith Tabullo De Robles, Francisca Fernández Valverde, Lucero Valladares Cisneros, Juana Hernández Villeda, Ayixon Sánchez-Reyes, María del Carmen Gutiérrez

**Affiliations:** 1 Center of Biotechnology Research, Autonomous University of the State of Morelos (UAEM), Cuernavaca, Mexico.; 2 Manuel Velasco Suárez National Institute of Neurology and Neurosurgery, México City, Mexico.; 3 Cátedras Conacyt-Institute of Biotechnology, National Autonomous University of Mexico (UNAM), Cuernavaca, Mexico.

**Keywords:** Centipede, Venom, Myotoxic activity

## Abstract

**Background:**

*Scolopendra polymorpha (S. polymorpha)* is a predatory centipede whose venom contains a multiplicity of biochemical effectors that can cause muscle damage and cumulative cell destruction in its prey. Despite previous investigations of *S. polymorpha* and other centipede venoms, there is a lack of information on the morphological and biochemical patterns elicited by their myotoxic effects. To elucidate these processes, this paper presents evidence of skeletal muscle damage, and alterations in key biochemical mediators that appear only after exposure to centipede venom.

**Methods:**

Venom was collected and fractionated using RP-HPLC; mouse *extensor digitorum longus* (EDL) muscle was exposed to whole venom and venom fractions to evaluate myotoxicity by means of creatine kinase (CK) - a muscle damage marker - activity measurements and histochemical analysis.

**Results:**

CK activity was higher in EDL muscle exposed to venom than in unexposed muscle. This increase was observed after 15 min of venom incubation, and remained stable up to 45 min. Venom-exposed EDL muscle showed signs of muscle damage including necrosis, loss of fascicular structure as well as mitochondrial accumulations and ragged red fibers (RRF), suggesting an impairment in the normal mitochondrial arrangement. Nicotinamide adenine dinucleotide (NADH) and cytochrome oxidase (COX) tests also indicate that respiratory complexes might be affected.

**Conclusion:**

Our results suggest a different biochemical composition of *S. polymorpha* venom, based on the different effects of four venom fractions on the cells tested, according to statistical evidence. Fractions F6 and F7 caused the most important alterations.

## Background

Centipedes or chilopods are venomous arthropods widely distributed throughout the world - except for Antarctica - comprising more than 3300 species and 5 orders, namely Scutigeromorpha, Lithobiomorpha, Craterostigmomorpha, Geophilomorpha and Scolopendromorpha [1,2]. They possess venomous glands located in the head, inside a pair of forcipules; the Scolopendromorpha genus includes those centipedes of medical importance since their bites to humans, however rare, cause local symptoms such as burning pain, edema and inflammation. There have been reports of headaches, anxiety, respiratory difficulties and even acute renal failure and multifocal neuropathy induced after drinking alcohol soaked with a specimen of *Scolopendra subspinipes mutilans,* currently used in Chinese traditional medicine [3-7]. On the other hand, recent studies confirm that centipede venoms are a rich and diverse source of novel compounds and structural scaffolds, such as antimicrobial peptides with potential biotechnological applications [8-10]. 

Despite their diversity, centipede venoms have not been as much studied as those of spiders, scorpions or snakes. Most of the reports on centipede venoms are focused on Asian species and only a few of them deal with their effects at a histological level [11,12]. Hence, there is very scarce information available on the potential myotoxic effects of centipede venom, the local tissue damage that it produces and the underlying mechanisms or chemical nature of its toxic components [6.^13^.^14^]. In a previous study [14] on the effects on the nociception of mice provoked by the venom of the common desert centipede, *Scolopendra polymorpha*, autochthonous to the northern and central regions of Mexico [1,15], we found evidence of morphological and biochemical alterations in skeletal muscle. Therefore, this study aimed to explore the*in vitro*myotoxic potential of*S. polymorpha* venom, looking for muscular damage signals, structural integrity modifications and cellular respiration impairing, to elucidate the basic cellular mechanisms associated with muscle pathology in general. In this paper we offer histological and biochemical evidence for localized muscle tissue damage such as loss of both fascicular structure and fiber polygonal shape, signs of necrosis, ragged fibers with certain accumulation of matter on the periphery of the fibers, suggesting possible damage to the electron transport chain in response to centipede venom. 

## Material and Methods

### Animals and Venom

Female CD-1 mice (20-25 g, 4-6 weeks old) were provided by the Animal House of the Institute of Biotechnology (IBT), National Autonomous University of Mexico (UNAM). Animals received food and water *ad libitum*. Centipedes (*S. polymorpha*) were collected in the state of Morelos, Mexico. Venom was obtained by manual stimulation of the venom claws, and collected by capillarity, placing a micropipette tip into a tube containing double-distilled water prior to quantification by the Lowry method [11] and maintained in storage at -20°C until further utilization.

### Reverse-Phase High Performance Liquid Chromatography (RP-HPLC)

Venom was fractionated by reverse-phase high performance liquid chromatography (RP-HPLC), in a C18 reverse-phase analytical column (Vydac, Hesperia, CA), using a linear gradient from 0% solvent A (0.12% trifluoroacetic acid, TFA, in water) to 60% solvent B (0.1% TFA in acetonitrile), at a flow rate of 1 mL/min for a 60-min run, according to González-Morales and collaborators [16]. Fractions were manually collected every 5 min., desalted, concentrated to dryness and stored at -20ºC for further experiments. 

### Sodium Dodecyl Sulfate-Polyacrylamide Gel Electrophoresis (SDS-PAGE)

Ten micrograms of each fraction were analyzed by SDS-PAGE (12% acrylamide gel) under non-reducing conditions [12]. Gels were stained with Coomassie blue (R-250, Bio-Rad, USA). The prestained molecular weight markers (MWM) used were myosin, β-galactosidase, bovine serum albumin, carbonic anhydrase, soybean trypsin inhibitor, lysozyme and aprotinin, ranging from 6.5 to 198 kDa (Prestained SDS-PAGE Standards, broad range, Bio-Rad, USA).

### ***In vitro* CK Activity Assay**

*Extensor digitorum longus* (EDL) muscle from mice was dissected, weighed and submerged in 3 mL of saline solution (SS) containing NaCl (135 mM), KCl (5 mM), CaCl_2_ (2 mM), MgCl_2_ (1 mM), NaHCO_3_ (15 mM), NaH_2_PO_4_ (1 mM) and glucose (11 mM), with constant bubbling (O_2_ and CO_2_) at 37°C, as described by Fuly et al. [13]. This solution was changed every 15 min for 45 min. Thereafter, the venom or venom fractions to be evaluated were added at a final concentration of 10 µg/mL. Aliquots of 100 µL were removed at different times (0, 15, and 45 min) throughout the assay. Negative control was conducted in the presence of the SS. At the end of the experiment, CK activity was measured in each aliquot using a commercial kit (Human Gesellschaft für Biochemica und Diagnostica mbH, Wiesbaden Germany). Briefly, 50 µL from each sample was solubilized in 1 mL of reactive solution, and optical density was measured at 340 nm at 0, 1, 2 and 3 min, to determine ΔA/min and obtain enzymatic activity in international units (U/L). Afterwards, the activity was normalized taking into account the weight (g) of each muscle sample.

### Histochemistry

EDL muscle used in the CK activity assays was frozen in isopentane previously cooled with liquid nitrogen. Transverse sections (7 µm thick) were cut using a cryostat (-19°C) and subsequently stained with either hematoxylin and eosin (H&E), or Gomori’s modified trichrome staining (MGT) and examined under light microscopy.

### Mitochondrial Impairment

Mitochondrial impairment was evaluated by the enzymatic response of respiratory chain complexes I and IV. 

*NADH assay*

Transverse 7-µm-thick sections of EDL muscle were incubated in a phosphate buffer (containing NaH_2_PO_4_ and Na_2_PO_4_ 0.2M, pH= 7.4) with nitro-blue tetrazolium (NBT, 4 mg) and NADH (10 mg). Slices were kept in the solution for 30 minutes at room temperature before mounting. Reduction of NBT yields the precipitation of a purple formazan compound at the sites of mitochondrial activity in the sarcoplasmic reticulum. This staining technique allows the identification of Type I fibers, which appear darker than Type II fibers. 

*COX assay*

Muscle sections were incubated at 37°C for 30 minutes in a phosphate buffer (with NaH_2_PO_4_ and Na_2_PO_4_ 0.2M, pH= 7.4) containing 10 mg of diaminobenzidine, then were mounted. Mitochondrial activity is evidenced by a brownish coloration; Type I fibers appear darker than Type II ones. 

### Statistical Analysis

Data were submitted to analysis of variance (ANOVA), considering a completely random design. ANOVA hypotheses were verified by the following tests: Kolmogorov-Smirnov to evaluate normality [20] and Levene’s to check variance homogeneity [14]. Multiple comparison of means was fulfilled through Fisher’s LSD test (least significant difference test). The significance level was established (α = 0.05) and STATISTICA v7.0 (StatSoft, Inc., Tulsa, US) was used for analyzing data. 

## Results

### Venom Fractionation

We obtained twelve venom fractions (F1 to F12) from RP-HPLC (Additional file 1, panel A) and selected fractions F4, F6, F7, and F8 for testing of their myotoxic effects on mouse tissue. These fractions were selected after several chromatographic runs on the basis of their protein content evaluated through SDS-PAGE (Additional file 1, panel B).

### ***In vitro* CK Activity**

**Figure 1.**
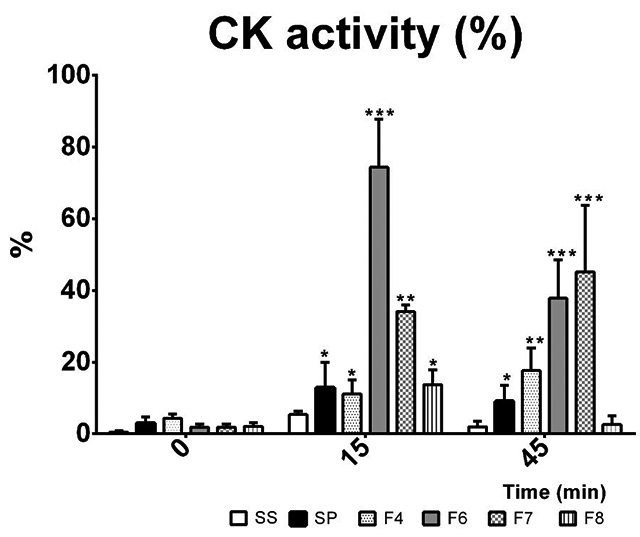
CK *in vitro* activity of whole venom and venom fractions. CK activity, expressed as a percentage, was measured before the addition of venom or venom fractions (time = 0 min) and 15 and 45 min after muscle exposure to venom or venom fractions. SS: saline solution; SP: whole venom (10 µg/mL); F4-F8: venom fractions (10 µg/mL each). n = 3; *statistical significance (p ˂ 0.05) between groups, ANOVA.

SP and fractions (F4, F6, F7) triggered CK activity in muscle incubated for 15 and 45 min, which is an indicator of early muscle damage, while SS did not cause a significant elevation (Figure 1). Although SP and the fractions tested showed statistically significant differences versus the SS control (ANOVA univariate tests of significance *P*_*value*_ <0.00002), the effects were more evident with the individual fractions. F6 accounted for almost 80% of the CK activity at 15 minutes (LSD test *P*_*value*_ = 0.00013), while F7 accounted for nearly 50% of the CK activity at 45 minutes (LSD test *P*_*value*_ = 0.002). The F4 and F8 fractions did not contribute significantly to the increase in CK. 

### ***S. polymorpha* Venom Induced Morphological Muscle Alterations and Severe Myotoxic Effects**

*Histological analysis*

**Figure 2.**
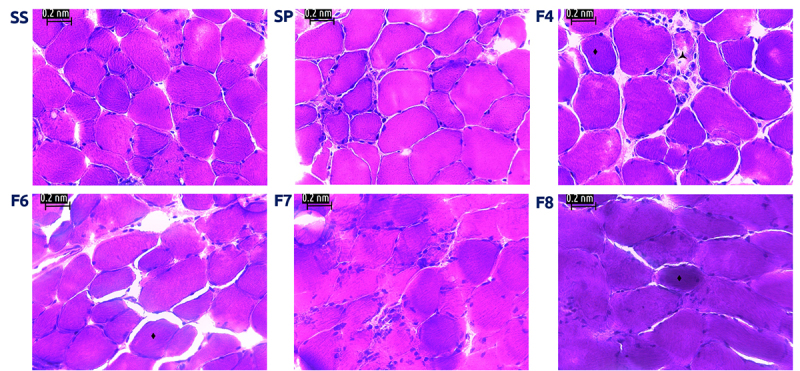
Light micrographs of mouse EDL muscle exposed to *S. polymorpha* whole venom or its fractions. (SS) Muscle incubated with saline solution. Normal fibers with polygonal shape, peripheral nuclei and conserved fascicular structure. Muscle incubated with whole venom (SP) or venom fractions F4, F6, F7 and F8, respectively; there are signs of necrosis (🟀), loss of fascicle architecture, hyperchromic round shaped fibers (🞞) and contracted cells (*). 400x; H&E stain.

**Figure 3.**
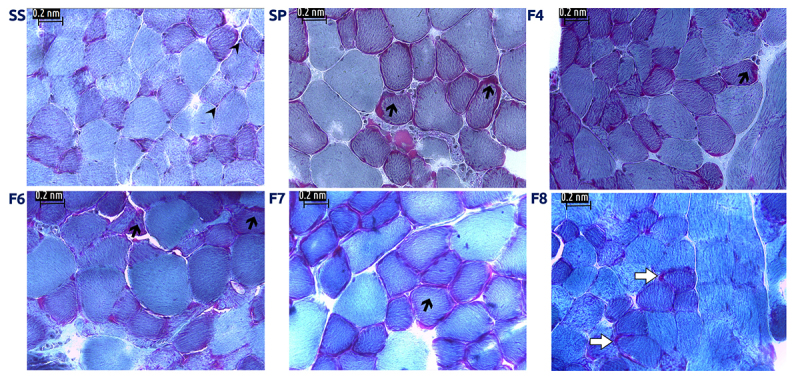
Mouse EDL muscle exposed to *S. polymorpha* whole venom or its fractions showed signs of mitochondrial damage. (SS) Muscle incubated with saline solution. Normal fibers with polygonal shape, peripheral nuclei and conserved fascicular structure. Muscle incubated with whole venom (SP) or venom fractions F4, F6, F7 and F8, respectively; some fibers show the appearance of ragged red fibers (arrows) associated to altered mitochondrial activity; peripheral nuclei can be observed (arrow heads). Areas of mitochondrial accumulation were also found (white arrows). 400x; MGM.

Muscle cross-sections showed not only loss of fascicle architecture, along with signs of necrosis in muscle exposed to *S. polymorpha* whole venom (SP) and fractions, but also the presence of dark-stained abnormal rounded fibers, whereas samples exposed to saline solution (SS) showed a higher level of conservation. Fiber cells were polygonal in shape, with peripheral nuclei, and were organized in fascicles (Figure 2). Furthermore, modified Gomori trichrome stain evidenced little conservation of muscle structure in treated cells, shown by the presence of “ragged red fibers” (RRF) (Figure 3).

We quantified the number of RRF per field in a total of 9 fields per condition. A statistical analysis (ANOVA) showed significant differences between samples exposed to both whole venom (SP) and venom fractions (F4, F6 and F7) versus the negative control (SSI) (p-value: 0.00; F: 45.3), with the exception of muscle incubated with fraction F8, whose effect was not different from that of SS. 

*Mitochondrial activity disruption signals induced by S. polymorpha venom*

**Figure 4.**
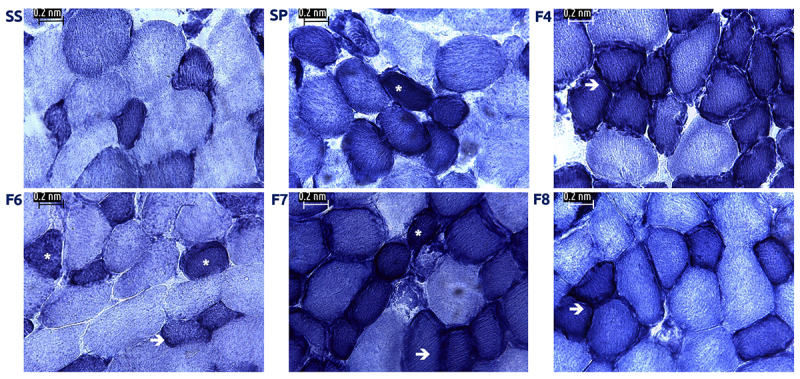
NADH enzymatic activity. (SS) Muscle incubated with saline solution. We observe a chess board-like distribution of fibers Type I (darker) and Type II (B, C, D, E, F). Muscle incubated with whole venom (SP) or venom fractions (F4 to F8); there are signs of mitochondrial alterations, such as excessive coloration (*, “blue” fibers). Areas of mitochondrial accumulation were also found (white arrows). 400x.

**Figure 5.**
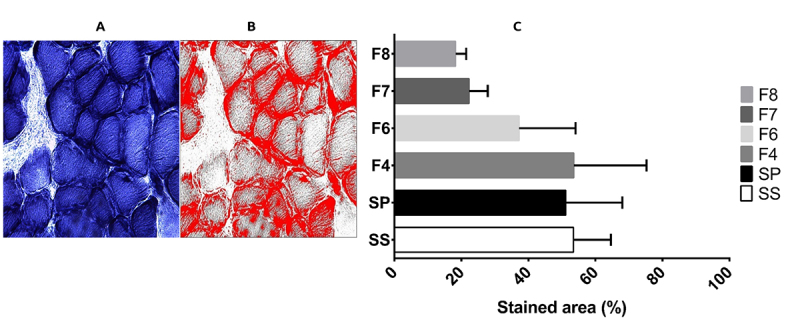
NADH activity quantification via the software FIJI. **(A)** Micrograph without filters, showing NADH activity. **(B)** Micrograph with filters to emphasize the areas of enzymatic activity, in red. Magnification: 400x. **(C)** NADH stained areas. After software treatment, a statistical analysis of the percentage of NADH stained areas was performed. *statistical significance (p ˂ 0.05).

**Figure 6.**
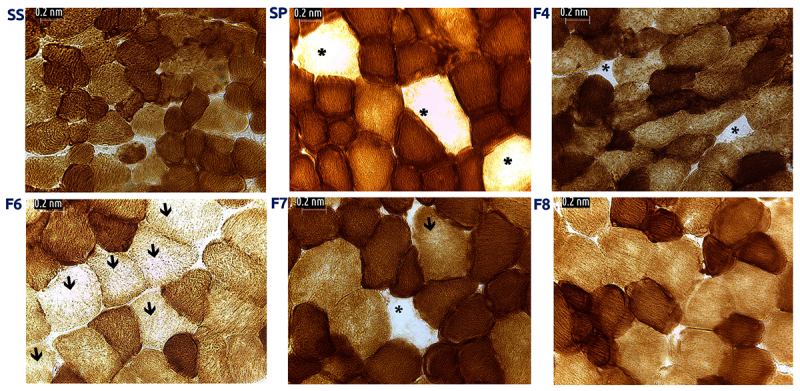
COX enzymatic activity. (SS) Muscle incubated with saline solution. Type I fibers appear in dark brown whereas Type II fibers are lighter. Muscle incubated with whole venom (SP) or venom fractions (F4 to F8); there are signs of mitochondrial alterations, evidenced by the presence of COX negative fibers (*) or areas with weakened reaction (arrows). 400x.

When testing NADH enzymatic activity we found the presence of ragged blue fibers, which stained darkest (Figure 4, arrows) in muscle treated with SP and fractions. In comparison, control slices showed a regular distribution of dark (Type I) and lighter (Type II) stained fibers. It is noteworthy that the presence of round shaped fibers was identifiable by H&E staining, which also showed abnormally elevated NADH activity, whereas some areas of diminished activity were found in larger (“swollen”) fibers. When we analyzed the stained area in treated and control cells we observed that the percentage of stained cells was associated with exposure to venom fractions, where F6, F7 and F8 showed the greatest effects (p = 0.002; ***p < 0.00001) (Figure 5). Upon evaluation of COX activity we found fibers with very weak reaction (*arrows*, Fig. 6E, venom fraction F6) and even COX-negative fibers that exhibited complete absence of reaction (asterisks in Fig. 6B, SP). In contrast, control fibers showed an even staining and were clearly distinguishable between types I (dark brown) and II fibers (light brown).

## Discussion

The cellular myotoxicity effects induced by snakes, scorpions and arachnid venoms have been widely reported in the scientific literature, mostly by their direct implications in acute envenomation in humans or domestic animals [2]. In contrast, centipede venoms have received less attention since there is a comparatively lower incidence of clinical cases. When evaluating the myotoxic effect of SP venom on mouse muscle tissue, we found it to induce not only structural alterations but also signs of mitochondrial disruption.

These could suggest that the contribution to muscle damage differed among the fractions due to differential enrichment of myotoxicity effectors in the fractions F6 and F7, the major contributors to CK increase, and also was evidenced by the number of RRF per field. 

Myotoxicity was evaluated *in vitro* by histochemistry and CK activity determination. 

The presence of ragged red fibers is a common phenomenon provoked by snake and arachnid bites, caused - in human fibers - by the accumulation of abnormal mitochondrial aggregates below the sarcolemma [15] (Figure 2). These mitochondrial aggregates suggest possible alterations in the overall mitochondrial function of cells (see Additional file 2). These post-exposure structural effects may be due to the release of early inflammation effectors as well as the action of enzymatic mediators that account for myotoxicity [17].

Since increases in CK levels indicate cell damage of skeletal muscle fibers, these results are highly consistent with tissue damage found in the histological analysis and confirm that the observed effects are a consequence of myotoxic activity by *S. polymorpha* venom. Malta et al. [6], found that the venom of other scolopendrid species caused direct hemolysis and fibrinolytic activity, often associated with the centipede’s ability to produce metalloproteases, phospholipases and hyaluronidase enzymes. We detected local inflammatory and cell structural compromised signals sooner after inoculation (15 min), compared with *Scolopendra viridicornis* studies [16] that noted the first inflammatory and histological reactions at 30 min after inoculation in a similar animal model. Furthermore, the myotoxic activity seems to be the result of concerted actions of more than one component in the venom, since five purified components presented no effects on CK activity compared with SP (Additional file 3), while at least two components (14 kDa and 42 kDa bands) correspond with the phospholipase activity reported by González *et al*. [18]. To the best of our knowledge, this is the first comprehensive report on tissue damage provoked by *S. polymorpha* venom.

We evaluated the integrity of respiratory chain complex I with NADH stain, which is also utilized to differentiate between types of muscle fibers, where Type I stain is darker due to higher mitochondrial activity, while Type II appears lighter and reveals abnormal mitochondrial aggregation or altered activity, such as lack of reaction. We identified the presence of fibers stained very darkly (Figure 4), consistent with our MGT findings of ragged red fibers which suggest that centipede venom affects respiratory chain complex I, since those blue fibers were only found in tissue exposed to venom or its fractions. 

The NADH enzymatic test allows us to confirm that muscle treated with centipede venom experienced several alterations, such as the presence of ragged blue fibers - which stained darkest - consistent with the presence of ragged red fibers (RRF) we found with MGT stain resembling histologic signatures for mitochondrial myopathies in humans. A lack of reaction implies the total disruption of complex I (Figure 4B-E).

On the other hand, COX activity further confirms mitochondrial damage. These findings suggest damage to mitochondrial complex IV, especially in Type II fibers, which is consistent with the damage we identified when we evaluated NADH activity. 

Histochemical analysis shows that the centipede venom produces distinctive changes among negative control and cells treated with venom fractions, in spite of the different techniques we used. These findings could also be associated with membrane damage, since it has been found that some spider venoms cause cell contraction, membrane disruption and apoptosis [19]. It is noteworthy that HeLa cultures exposed to*S. polymorpha*whole venom exhibit similar responses [20], suggesting that there is a compositional regularity in the biochemical determinants contained in the venom of these centipedes, which are responsible for the myotoxic effects. Despite the fact that *S. polymorpha*venom proteins are renewed asynchronously [21], the myotoxic effects on different cells seem to be a consequence of the selection and evolution of this predatory and venomous animal.

## CONCLUSION

The study of animal venoms has contributed demonstrable benefits to the biomedical field. In spite of increasing evidence of the importance of centipedes as a reservoir of novel proteins and enzymes, their venoms are amongst the least studied ones in comparison to snakes and other arthropods, such as scorpions and spiders. Through the present study, we demonstrate that, in mice, the myotoxic effects of *S. polymorpha* venom readily manifest within a short period of time after exposure, causing severe structural and biochemical damage to skeletal muscle, which is expected when studying the venom of a predatory animal, whose components have evolved to affect a wide range of prey. 

### Abbreviations

ANOVA: analysis of variance; CEIB: Centro de Investigación en Biotecnología; CK: creatine kinase; CONACyT: Consejo Nacional de Ciencia y Tecnología; COX: cyclooxygenase; H&E: hematoxylin and eosin stain; IBT: Instituto de Biotecnología; LSD test: least significant difference test; MGT: Gomori’s modified trichrome stain; MWM: molecular weight markers; NADH: nicotinamide adenine dinucleotide (NAD) + hydrogen (H); 

NBT: nitro-blue tetrazolium; RP-HPLC: reverse-phase high performance liquid chromatography; RRF: ragged red fibers; *S. polymorpha: Scolopendra polymorpha*; SDS-PAGE: sodium dodecyl sulfate-polyacrylamide gel electrophoresis; SP: *S. polymorpha* venom; SS: saline solution; TFA: trifluoroacetic acid; UAEM: Universidad Autónoma del Estado de Morelos; UNAM: Universidad Nacional Autónoma de México.

## Supplementary material

The following online material is available for this article:

Additional file 1. Fractionation of *S. polymorpha* venom. **(A)** RP-HPLC profile. Crude venom was separated using a C18 column in RP-HPLC. Linear gradient from 0% buffer A (TFA 0.12% in H_2_O) to 60% buffer B (TFA 0.1% in ). Flow: 1 mL/min. Optical density was monitored at 230 nm. **(B)** SDS-PAGE of RP-HPLC fractions. Analysis performed under non-denaturing conditions. MWM: molecular weight markers; SP: *S. polymorpha* whole venom; F1-F11: venom fractions. Coomasie blue stain.

Additional file 2. Transmission electron microscopy (TEM) images showing mitochondrial alterations in mice skeletal muscle: **(A)** SS incubated muscle. Cross section; glycogen granules (circle) and normal mitochondria (M) can be observed. Z: Z-line. 25000x. **(B)** SP incubated muscle. Cross section. Altered mitochondria are shown (arrows). 25000x. **(C-D)** F4 incubated muscle. Cross section with normal (M) and altered mitochondria (arrow). 30000x and 60000x, respectively. **(E)** F6 incubated muscle. There are signs of basement membrane detachment (arrow). 30000x. **(F)** Mitochondria accumulation. Cross sections of F6 incubated muscle show areas of mitochondrial accumulation (ma); Z: Z-line. 60000x. **(G)** Nuclear alterations found in F6-incubated muscle. In addition to mitochondrial accumulation, there are abnormal nuclei (arrows). 15000x. **(H-I)** F7 incubated muscle. Mitochondria with altered cristae are shown (arrows). 60000x and 100000x.

Additional file 3. CK *in vitro* activity of electroeluted bands. CK activity, expressed as a percentage, was measured before the addition of venom or electroeluted bands (time = 0 min) and at 15 and 45 min after muscle exposure to venom or venom fractions. SS: saline solution; SP: whole venom; 9 kDa-56 kDa: electroeluted bands. Average values ± SEM are shown. *Statistical significance (p ˂ 0.05).
