# Supplementary material for: Mitochondrial activity disruption and local muscle damage induced in mice by Scolopendra polymorpha venom
Source: J Venom Anim Toxins Incl Trop Dis. 2020 May 29;26:e20190079. doi: 10.1590/1678-9199-JVATITD-2019-0079 (PMC7269145; doi:10.1590/1678-9199-JVATITD-2019-0079)
Supplement: Additional file 1. [file 1678-9199-jvatitd-26-e20190079-s1.pdf]

Supplementary material to: Mitochondrial activity disruption and local muscle damage induced in mice by *Scolopendra polymorpha* venom

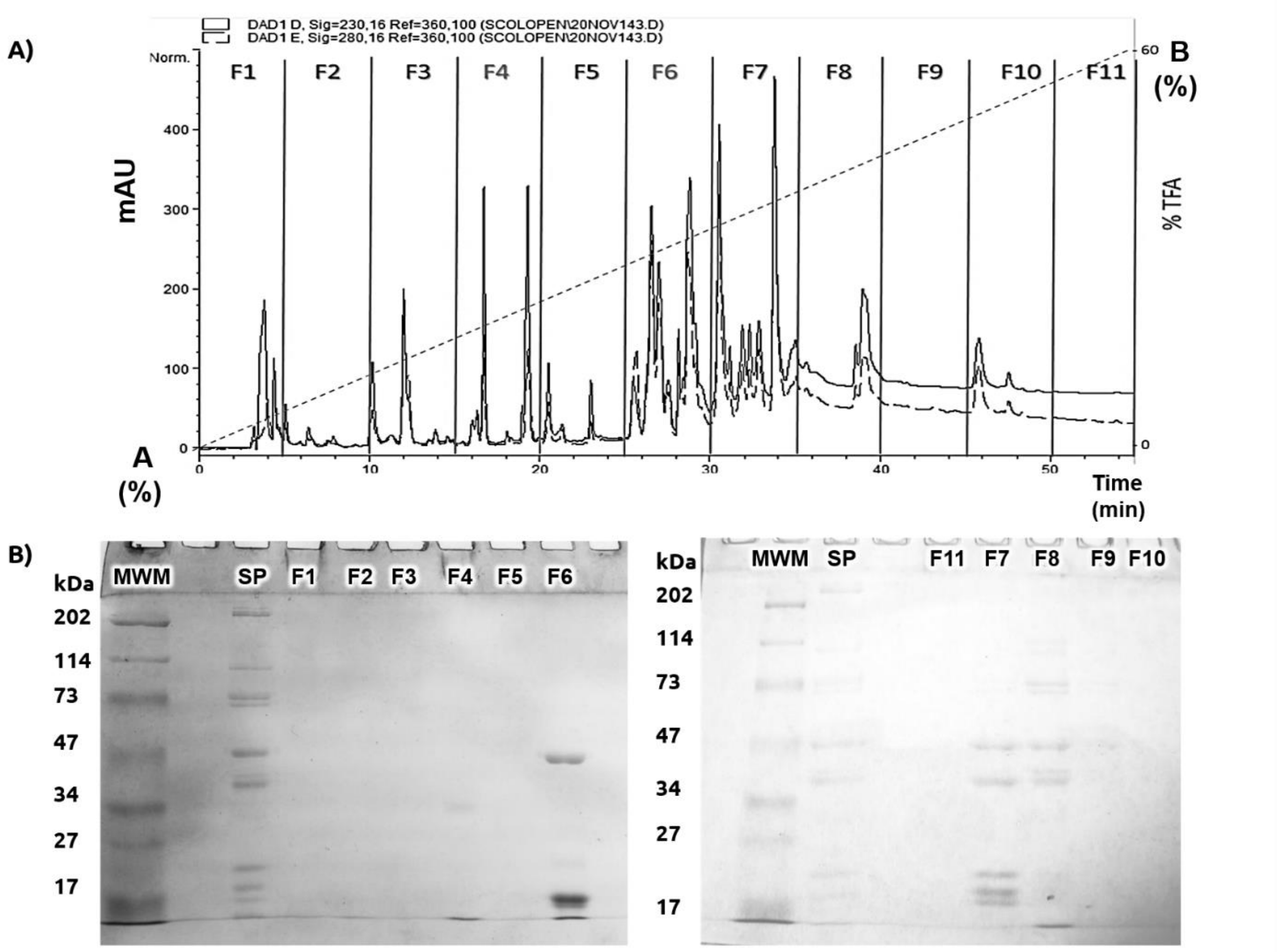

Additional file 1. Fractionation of *S. polymorpha* venom. **(A)** RP-HPLC profile. Crude venom was separated using a C18 column in RP-HPLC. Linear gradient from 0% buffer A (TFA 0.12% in H_2O) to 60% buffer B (TFA 0.1% in). Flow: 1 mL/min. Optical density was monitored at 230 nm. **(B)** SDS-PAGE of RP-HPLC fractions. Analysis performed under non-denaturing conditions. MWM: molecular weight markers; SP: *S. polymorpha* whole venom; F1-F11: venom fractions. Coomasie blue stain.
