# Supplementary material for: Mitochondrial activity disruption and local muscle damage induced in mice by Scolopendra polymorpha venom
Source: J Venom Anim Toxins Incl Trop Dis. 2020 May 29;26:e20190079. doi: 10.1590/1678-9199-JVATITD-2019-0079 (PMC7269145; doi:10.1590/1678-9199-JVATITD-2019-0079)
Supplement: Additional file 2. [file 1678-9199-jvatitd-26-e20190079-s2.pdf]

Supplementary material to: Mitochondrial activity disruption and local muscle damage induced in mice by *Scolopendra polymorpha* venom

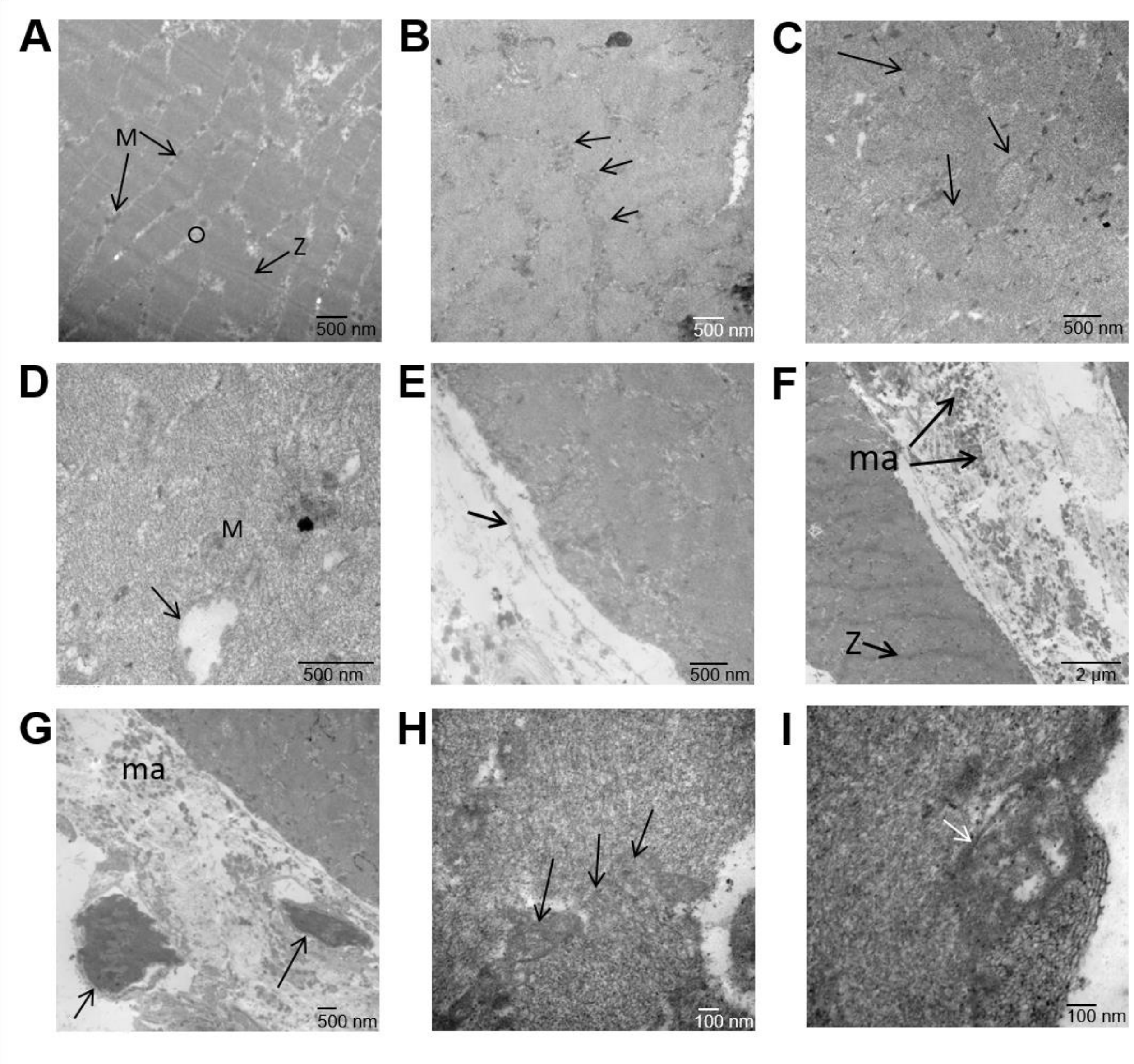

Additional file 2. Transmission electron microscopy (TEM) images showing mitochondrial alterations in mice skeletal muscle. **(A)** SS incubated muscle. Cross section; glycogen granules (circle) and normal mitochondria (M) can be observed. Z: Z-line. 25000x. **(B)** SP incubated muscle. Cross section. Altered mitochondria are shown (arrows). 25000x. **(C-D)** F4 incubated muscle. Cross section with normal (M) and altered mitochondria (arrow). 30000x and 60000x, respectively. **(E)** F6 incubated muscle. There are signs of basement membrane detachment (arrow). 30000x. **(F)** Mitochondria accumulation. Cross sections of F6 incubated muscle show areas of mitochondrial accumulation (ma); Z: Z-line. 60000x. **(G)** Nuclear alterations found in F6-incubated muscle. In addition to mitochondrial accumulation, there are abnormal nuclei (arrows). 15000x. **(H-I)** F7 incubated muscle. Mitochondria with altered cristae are shown (arrows). 60000x and 100000x.
