# Supplementary material for: Mitochondrial activity disruption and local muscle damage induced in mice by Scolopendra polymorpha venom
Source: J Venom Anim Toxins Incl Trop Dis. 2020 May 29;26:e20190079. doi: 10.1590/1678-9199-JVATITD-2019-0079 (PMC7269145; doi:10.1590/1678-9199-JVATITD-2019-0079)
Supplement: Additional file 3. [file 1678-9199-jvatitd-26-e20190079-s3.pdf]

Supplementary material to: Mitochondrial activity disruption and local muscle damage induced in mice by *Scolopendra polymorpha* venom

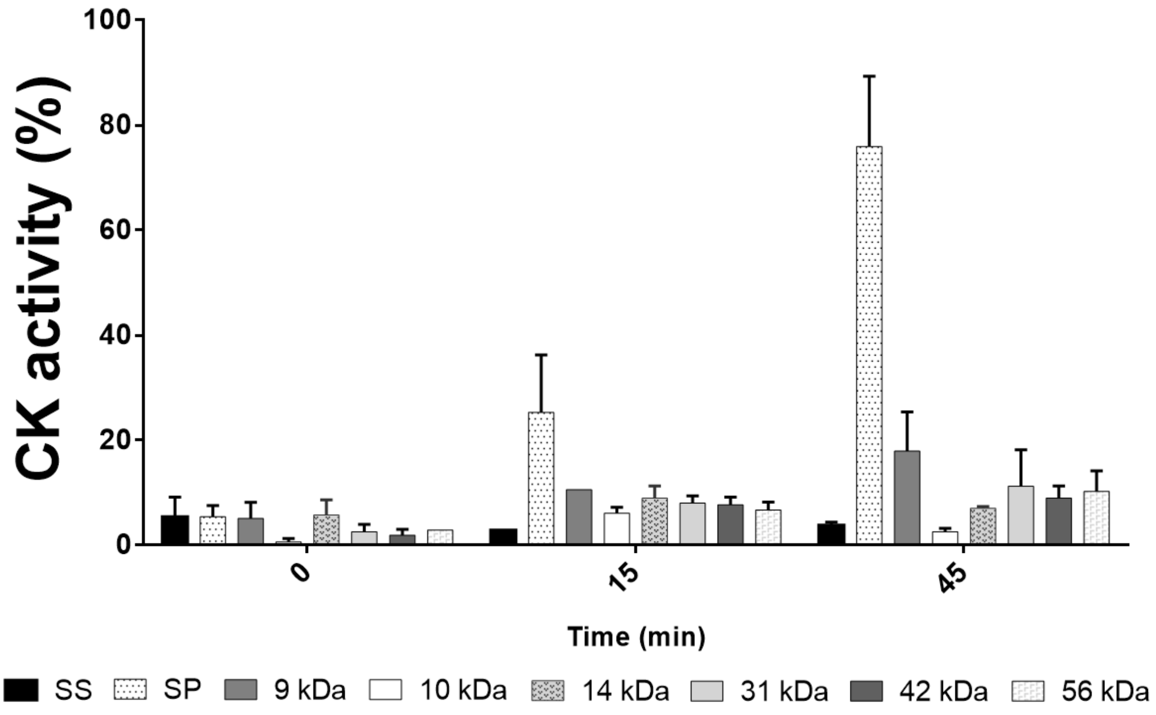

Additional file 3. CK *in vitro* activity of electroeluted bands. CK activity, expressed as a percentage, was measured before the addition of venom or electroeluted bands (time = 0 min) and at 15 and 45 min after muscle exposure to venom or venom fractions. SS: saline solution; SP: whole venom; 9 kDa-56 kDa: electroeluted bands. Average values ± SEM are shown. *Statistical significance ($p < 0.05$).
